# Prognostic Models of Mortality Following First‐Ever Acute Ischemic Stroke: A Population‐Based Retrospective Cohort Study

**DOI:** 10.1002/hsr2.70445

**Published:** 2025-02-13

**Authors:** Mustapha Mohammed, Hadzliana Zainal, Siew Chin Ong, Balamurugan Tangiisuran, Fatimatuzzahra Abdul Aziz, Norsima N. Sidek, Abubakar Sha'aban, Umar Idris Ibrahim, Surajuddeen Muhammad, Irene Looi, Zariah A. Aziz

**Affiliations:** ^1^ QU Health Qatar University Doha Qatar; ^2^ School of Pharmaceutical Sciences Universiti Sains Malaysia Pulau Pinang Malaysia; ^3^ Clinical Research Center Hospital Sultanah Nur Zahirah Terengganu Malaysia; ^4^ Division of Population Medicine Cardiff University Cardiff Wales; ^5^ Faculty of Pharmacy Universiti Sultan Zainal Abidin Kuala Terengganu Malaysia; ^6^ Faculty of Veterinary Medicine Ahmadu Bello University Kaduna Nigeria; ^7^ Clinical Research Center Hospital Seberang Jaya Pulau Pinang Malaysia

**Keywords:** acute ischemic stroke, calibration, discrimination, mortality, prognostic model, validation

## Abstract

**Background and Aims:**

There is a lack of population‐based studies focusing on guideline‐based prognostic models for stroke. This study aimed to develop and validate a prognostic model that predicts mortality following a first‐ever acute ischemic stroke.

**Methods:**

The study included 899 adult patients ( ≥ 18 years) with confirmed diagnosis of first‐ever acute ischemic stroke enrolled in the Malaysian National Stroke Registry (NSR) from January 2009 to December 2019. The primary outcome was mortality within 90 days post‐stroke (266 events [29.6%]). The prognostic model was developed using logistic regression (75%, *n* = 674) and internally validated (25%, *n* = 225). Model performance was assessed using discrimination (area under the curve (AUC]) and calibration (Hosmer‐Lemeshow test [HL]).

**Results:**

The final model includes factors associated with increased risk of mortality, such as age (adjusted odds ratio, aOR 1.06 [95% confidence interval, CI 1.03, 1.10; *p* < 0.001]), National Institutes of Health Stroke Scale (NIHSS) score aOR 1.08 (95% CI 1.08, 1.13; *p *= 0.004), and diabetes aOR 2.29 (95% CI 1.20, 4.37; *p* = 0.012). The protective factors were antiplatelet within 48 h. aOR 0.40 (95% CI 0.19, 0.81; *p* = 0.01), dysphagia screening aOR 0.30 (95% CI 0.15, 0.61; *p* = 0.001), antiplatelets upon discharge aOR 0.17 (95% CI 0.08, 0.35; *p *< 0.001), lipid‐lowering therapy aOR 0.37 (95% CI 0.17, 0.82; *p* = 0.01), stroke education aOR 0.02 (95% CI 0.01, 0.05; *p* < 0.001) and rehabilitation aOR 0.08 (95% CI 0.04, 0.16; *p* < 0.001). The model demonstrated excellent performance (discrimination [AUC = 0.94] and calibration [HL, *X* ^2^ *p *= 0.63]).

**Conclusion:**

The study developed a validated prognostic model that excellently predicts mortality after a first‐ever acute ischemic stroke with potential clinical utility in acute stroke care decision‐making. The predictors could be valuable for creating risk calculators and aiding healthcare providers and patients in making well‐informed clinical decisions during the stroke care process.

## 1 Background

Stroke, the second leading cause of mortality and disability globally, has seen a significant increase in its burden, with 16 million new cases and over six million deaths annually [1]. Developing countries bear the majority of this burden, accounting for stroke‐related deaths ( ~ 75%) and disability‐adjusted life years (DALYs) ( ~ 81.0%) [2]. Ischemic stroke represents about 75%–85% of all stroke cases, and approximately 75% are first‐time [3]. Asia, home to over half of the world's population, faces a significant stroke burden [4]. In Malaysia, stroke ranks as the third‐leading cause of death and the second‐leading cause of disability [5]. Due to an aging population, the incidence of stroke in the country is expected to increase sharply over the next two decades [6].

Studies show that the highest mortality risk following a stroke occurs within the first 30 days, with an average annual mortality rate of about 10% after that [7]. Approximately one‐third of stroke patients do not survive beyond 3 years [8]. In Malaysia, studies have reported an overall mortality rate of 37%, with most deaths occurring within the first month [9, 10]. Factors such as deterioration of the Glasgow Coma Scale (GCS), middle cerebral artery infarction, atrial fibrillation (AF), and diabetes are linked with rising mortality after stroke [11]. However, the type of stroke and care process could play a role in both short and long‐term prognosis.

Clinical guidelines offer evidence‐based recommendations for diagnosing, preventing, and treating diseases [12]. In Malaysia, the latest clinical practice guideline (CPG) for ischemic stroke was released in 2020 [13, 14, 15]. The CPG recommended key performance indicators incorporated into the Malaysian National Stroke Registry (NSR) to evaluate stroke care quality. These indicators include thrombolysis, antithrombotic therapies, dysphagia screening, lipid‐lowering therapy, deep vein thrombosis (DVT) prophylaxis, anticoagulation for AF, stroke education, and rehabilitation.

Clinical prediction models analyze patient data and treatment processes to estimate the likelihood of future events. These models are instrumental in stroke care, helping clinicians set goals and make informed decisions based on predicted outcomes [16, 17]. Although many prognostic models exist, most are not stroke‐specific, lack generalizability, and have limited clinical utility, especially for long‐term care [18]. Examples include the Framingham Score [19], QRISK [20], and EURO‐SCORE [21]. For a risk score to be valid, it must be accurate and generalizable [18, 22]. Recent data on stroke outcome prediction indicate that models based solely on clinical criteria have limited classification power [23]. However, no single prognostic model is universally applicable across all stroke subgroups, especially for the first acute ischemic stroke. Therefore, this study aimed to develop and validate guideline‐guided prognostic models for predicting mortality in patients with first‐ever acute ischemic stroke.

## 2 Methods

### 2.1 Study Design

This was a multi‐centered retrospective cohort study aimed at developing and validating a guideline‐guided prognostic model for mortality following a first‐ever acute ischemic stroke in patients treated at the Hospitals recognized by the Ministry of Health (MOH) in Malaysia from January 2009 to December 2019. The study was reported following the recommendations of the Transparent Reporting of a multivariable prediction model for Individual Prognosis Or Diagnosis (TRIPOD) [24], the Strengthening the Reporting of Observational Studies in Epidemiology (STROBE) [25], and the Guidelines for Reporting of Statistics for Clinical Research [26].

### 2.2 Study Setting

The study included patients with first‐ever acute ischemic stroke registered in the Malaysian National Stroke Registry (NSR), a multiethnic, multicentered, and population‐based registry established in 2009 under the National Neurology Registry (NNeuR), Ministry of Health, Malaysia [13, 27]. Malaysia, located in Southeast Asia, consists of Peninsular Malaysia and East Malaysia. The country has 13 states with a population of approximately 33 million as of 2021, and mainly the Malays, Chinese, and Indians [6].

### 2.3 Study Population

The study included adult patients (aged 18 years and above) with a confirmed diagnosis of first‐ever acute ischemic stroke who were enrolled in the NSR between January 1, 2009, and December 31, 2019, and had 3 months of follow‐up data after hospital discharge. A first‐ever acute ischemic stroke is defined as the initial occurrence of an index stroke with a measurable neurological deficit lasting more than 24 h. due to presumed ischemic etiology [28, 29]. Patients with other stroke types, such as transient ischemic attack (TIA) and hemorrhagic stroke, those with recurrent stroke, those with terminal illnesses like cancer, those with significant missing data ( > 50%), and those without follow‐up data were excluded.

### 2.4 Study Outcomes

The primary prognostic outcome assessed was 90‐day mortality post‐stroke event, recorded as death, alive, or censored. A 90‐day follow‐up was chosen because significant stroke outcomes are typically reported within 3 months after discharge [30]. Moreover, the short‐term prognosis of cardioembolic stroke and atherothrombotic stroke (e.g., AF and left ventricular thrombus) is poor compared with other ischemic stroke subtypes [31].

### 2.5 Data Collection

The lead researchers (MM, HZ, and SCO) were involved in the data collection, cleaning, and management. The study collected data on various variables, including socio‐demographic characteristics (e.g., age, gender, ethnicity), clinical characteristics (e.g., GCS for consciousness assessment) [32], Oxfordshire Community Stroke Project (OCSP) classification for stroke subtypes [33], National Institutes of Health Stroke Scale (NIHSS) for stroke severity [34], comorbidities (e.g., diabetes, hypertension, hyperlipidemia), and application of the nine key performance indicators (KPIs) of the acute ischemic stroke management guideline. The KPIs include thrombolysis, antiplatelets therapy within 48 h, dysphagia screening, lipid‐lowering therapy, antiplatelets therapy upon discharge, DVT prophylaxis, anticoagulation for AF, stroke education (e.g., self‐care education, medication counseling), and rehabilitation (e.g., physical therapy care plan) [27].

### 2.6 Model Development

Accurate and reliable data on key predictors are paramount for effective model development; hence, selecting an appropriate study design is essential [34]. This study employs a logistic regression approach, as it enables multivariable modeling of a binary outcome variable (e.g., dead vs. alive). Logistic regression estimates coefficients for each predictor in the final model, capturing their contributions to the outcome [35].

#### 2.6.1 Univariable Analysis

A simple logistic regression was first used to screen independent variables (including sociodemographic and clinical characteristics) for their association with 90‐day mortality for the first acute ischemic stroke. Variables were considered for inclusion in the multiple logistic regression (MLR) analysis if they were significant at *p* < 0.25 or clinically relevant, as suggested in previous association studies [36, 37, 38].

#### 2.6.2 Multivariable Analysis

The MLR was conducted using the Backward LR method. Multicollinearity and interactions among variables were assessed to ensure the stability of the model. Model adequacy was evaluated using the Hosmer‐Lemeshow test and the Omnibus goodness‐of‐fit test. Additionally, the explanatory power of the model was assessed by Cox and Snell's *R*² and Nagelkerke's *R*² [39].

### 2.7 Model Validation

Internal validation is critical for ensuring the model's performance is not overly optimistic [40, 41]. Overfitting is especially likely when the number of outcomes is relatively small compared to the number of candidate predictors [42]. Accordingly, internal validation helps quantify overfitting risk and determine the reproducibility of the model within the same population. Resampling methods, such as cross‐validation and bootstrapping, are commonly employed to perform internal validation. In practice, the degree of optimism in a model's performance can be measured by comparing its performance metric (e.g., the c‐index) in bootstrap samples with that in the original sample. The study model performance was evaluated through calibration and discrimination methods [36].

#### 2.7.1 Calibration

Calibration describes how closely the model's predicted probabilities align with observed outcomes. This study assessed calibration using the Hosmer‐Lemeshow (H‐L) goodness‐of‐fit test. Individual risk scores were calculated, and patients were grouped into risk quintiles due to a relatively small number of observed events. Calibration plots, which visually illustrate mean predicted probabilities against the observed outcome proportions, can also be used. Preferably, a well‐calibrated model exhibits an intercept of 0 and a slope of 1 [41].

#### 2.7.2 Discrimination

Discrimination reflects the model's ability to distinguish between different outcome states. This study assessed the model discrimination using the receiver operating characteristic (ROC) curve, commonly used to plot sensitivity against specificity at varying risk thresholds. The area under the ROC curve (AUC) quantifies how effectively the model differentiates between outcomes [41, 43].

### 2.8 Sampling and Sample Size

All adult patients with a confirmed diagnosis of first‐ever acute ischemic stroke enrolled in the Malaysian National Stroke Registry were included in the study. For the minimum sample size required, prognostic studies recommend a minimum sample size of approximately 10 events per predictor variable (EPV) to develop binary prediction models and prevent overfitting [44].

#### 2.8.1 Sample Size Calculations

Sample size, *n* = 10 events per predictor variable (EPV).

Number of predictor variables: 10.

Mortality cases, n_m_ = 10 × 10 = 100.

Sample size, *n* = 10 events per predictor variable (EPV) = 100.

### 2.9 Statistical Analysis

Statistical analyses were performed following the Statistical Analyses and Methods in the Published Literature (SAMPL) guidelines [45]. The patients' data collected in Microsoft Excel format were imported into IBM SPSS Statistics for Windows, version 26.0 (IBM Corp., Armonk, NY) for statistical data analysis. Missing data were handled using multiple imputation methods. Descriptive statistics summarized categorical variables as frequencies and percentages, while continuous variables were summarized as means (standard deviations, SD).

Binary logistic regression was used to identify the independent factors (predictors) significantly associated with mortality (dead or alive). The univariable analysis was initially conducted to identify significant variables for inclusion in the multivariable logistic regression (MLR). The backward elimination method was used for MLR, and multicollinearity was assessed via a correlation matrix. Model fit assumptions were evaluated using the Hosmer‐Lemeshow and Omnibus tests. The final model was presented as adjusted odds ratios (aOR) with 95% confidence intervals (CIs) and *p*‐values. A two‐sided *p* < 0.05 was considered statistically significant.

## 3 Ethics Approval

All study procedures involving human participants followed the Declaration of Helsinki 1964 [46], and its amendments. The study was registered with the Malaysian National Medical Research Register (NMRR) (Reference No. NMRR‐19‐3846‐52122[IIR]) and approved by the Medical Research and Ethics Committee (MREC), Ministry of Health (MOH), Malaysia (Reference No. KKM/NIHSEC/P20‐307[6]). All study phases, including access to the stroke registry data and medical records, have been authorized by the National Institutes of Health (NIH) for Conducting Research in the Ministry of Health, Malaysia. The study was registry‐based using deidentified data without direct contact with the patients. Thus, informed consent was not applicable.

## 4 Results

### 4.1 Sociodemographic and Clinical Characteristics of the Study Population

A total of 899 eligible patients with first‐ever acute ischemic stroke were included in the study. The patients' average ( ± SD) age was 60.1 ± 10.8, mostly 60 years and above. Most of the patients were males (60.8%), belonging to the Malay ethnic group (73.1%), followed by Chinese (14.2%) and Indians (2.4%). The OCSP classified the patients into mostly LACI (39.0%) and PACI (29.0%). Others were TACI (15.1%), POCI (11.6%), and unclassified (5.2%). The patients had an average ( ± SD) GCS score of 14.1 ± 2.1, a scale used to assess the level of consciousness, and were grouped into mostly mild (13–15 points) (83.2%), followed by moderate (9–12 points) (14.1%) and severe (1–8 points) (2.7%). Furthermore, the patients had an average NIHSS score ( ± SD) of 7.9 (7.3), classified the patients as having majorly mild (1–4) (34.4%) and (moderate 5–15) (34.4%) strokes. Other NIHSS scores recorded were none (0) (7.9%), moderate‐severe (16–20) (7.7%), and severe (21–42) (7.5%). The study observed fewer patients arriving at the hospital within 3 h of stroke onset (26.1%) with an average ( ± SD) hospital stay of 6.3 ± 6.1 days. The patients' common risk factors and comorbid diseases were hypertension 618 (68.7%) and diabetes 421 (46.8%). Another important risk factor was hyperlipidemia 192 (23.7%). The primary outcome measured at the end of the study was all‐cause mortality 266 (29.6%). The summary of the sociodemographic and clinical characteristics of the study population is shown in Table 1.

**Table 1:** Sociodemographic and clinical characteristics of the study population.

Variable	Total, *n* (%) (*N* = 899)	Mortality, *n* (%) (*N* = 266)
Age (years)	61.1 (10.8)^a^	
Gender		
Female	352 (39.2)	108 (40.6)
Male	547 (60.8)	158 (59.4)
Ethnicity		
Malay	657 (73.1)	219 (82.3)
Chinese	128 (14.2)	27 (10.2)
India	22 (2.4)	11 (4.1)
Others	92 (10.2)	9 (3.4)
OCSP classification		
TACI	136 (15.1)	38 (14.3)
PACI	261 (29.0)	104 (39.1)
LACI	351 (39.0)	80 (30.1)
POCI	104 (11.6)	26 (9.8)
Unclassified	47 (5.2)	18 (6.8)
GCS score	14.1 (2.1)^a^	
Mild	748 (83.2)	214 (80.5)
Moderate	127 (14.1)	45 (16.9)
Severe	24 (2.7)	7 (2.6)
NIHSS score	7.9 (7.3)^a^	
None	71 (7.9)	14 (5.3)
Mild	309 (34.4)	80 (30.1)
Moderate	383 (42.6)	131 (49.2)
Moderate‐Severe	69 (7.7)	14 (5.3)
Severe	67 (7.5)	27 (10.2)
Risk factors		
Hypertension	618 (68.7)	192 (72.2)
Diabetes	421 (46.8)	156 (58.6)
Hyperlipidemia	192 (21.4)	68 (25.6)
Ischemic heart disease	87 (9.7)	30 (11.3)
Atrial fibrillation	28 (3.1)	8 (3.0)
Arrival time (≤ 3 h)	235 (26.1)	58 (21.8)

Abbreviations: GSC, Glasgow coma scale; LACI, lacunar infarct; mRS, modified Rankin scale; NIHSS, National Institute of Health Stroke Scale; OCSP, Oxfordshire community stroke project; PACI, partial anterior circulation infarct; POCI, posterior circulation infarct; SD, standard deviation; TACI, total anterior circulation infarct.

^a^ Mean (Standard Deviation, SD).

### 4.2 Predictors of Mortality Following First‐Ever Acute Ischemic Stroke: Univariable Model

The univariable analysis identified several factors significantly associated with mortality following the first‐ever acute ischemic stroke, as shown in Table 2. Factors associated with an increased mortality risk include age (odds ratio [OR] 1.03 [95% confidence interval [CI] 1.02, 1.05; *p* < 0.001]), PACI vs. TACI (OR 1.71, 95% CI 1.09, 2.68; *p *= 0.02), NIHSS score (OR 1.03, 95% CI 1.01, 1.05; *p *= 0.005), diabetes (OR 1.97, 95% CI 1.47, 2.63; *p *< 0.001), and hyperlipidemia (OR 1.41, 95% CI 1.01, 1.98; *p* = 0.05). Conversely, factors associated with a decreased risk of mortality include being Chinese versus Malay (OR 0.54, 95% CI 0.34, 0.84; *p *= 0.007), Others versus Malay (OR 0.22, 95% CI 0.11, 0.44; *p *< 0.001), antiplatelet therapy within 48 h. (OR 0.16, 95% CI 0.12, 0.22; *p *< 0.001), dysphagia screening (OR 0.11, 95% CI 0.08, 0.15; *p *< 0.001), antiplatelet therapy upon discharge (OR 0.10, 95% CI 0.01, 0.14; *p *< 0.001), lipid‐lowering agent (OR 0.51, 95% CI 0.37, 0.72; *p *< 0.001), stroke education (OR 0.02, 95% CI 0.01, 0.03; *p *< 0.001), and rehabilitation (OR 0.08, 95% CI 0.56, 0.11; *p *< 0.001).

**Table 2:** Predictors of mortality following first‐ever acute ischemic stroke: univariable model.

Variables	OR (95% CI)	*p*‐value
Age (Years)	1.03 (1.02–1.05)	< 0.001
Gender (Female)	1.09 (0.81–1.46)	0.57
Ethnicity		
Malay	1	1
Chinese	0.54 (0.34–0.84)	0.007
India	2.00 (0.85–4.69)	0.11
Others	0.22 (0.11–0.44)	< 0.001
Stroke subtype		
TACI	1	1
PACI	1.71 (1.09–2.68)	0.02
LACI	0.76 (0.49–1.19)	0.24
POCI	0.86 (0.48–1.54)	0.61
Unclassified	1.60 (0.80–3.22)	0.19
GCS score (1–15)	0.95 (0.89–1.02)	0.13
NIHSS score (0–42)	1.03 (1.01–1.05)	0.005
Arrival time (≤ 3 h)	0.73 (0.52–1.02)	0.07
Risk factors		
Hypertension	1.26 (0.92–1.73)	0.15
Diabetes	1.97 (1.47–2.63)	< 0.001
Hyperlipidemia	1.41 (1.01–1.98)	0.05
Ischemic heart disease	1.29 (0.81–2.05)	0.29
Atrial fibrillation (AF)	0.95 (0.41–2.19)	0.91
KPIs adherence		
Thrombolysis	0.43 (0.09–1.95)	0.27
Antiplatelet within 48 h.	0.16 (0.12–0.22)	< 0.001
Dysphagia screening	0.11 (0.08–0.15)	< 0.001
DVT prophylaxis	1.01 (0.73–1.40)	0.97
Antiplatelet upon discharge	0.10 (0.07–0.14)	< 0.001
Anticoagulant for AF	0.92 (0.57–1.50)	0.74
Lipid‐lowering agent	0.51 (0.37–0.72)	< 0.001
Stroke education	0.02 (0.01–0.03)	< 0.001
Rehabilitation	0.08 (0.56–0.11)	< 0.001

*Note:* Statistical significance at *p* < 0.05 using simple logistic regression.

Abbreviations: CI, confidence interval; GSC, Glasgow Coma scale; KPIs, key performance indicators; LACI, lacunar infarct; mRS, modified Rankin scale; NIHSS: National Institute of Health Stroke Scale; OCSP, Oxfordshire community stroke project; OR, crude odds ratio; PACI, partial anterior circulation infarct; POCI, posterior circulation infarct; SD, standard deviation; TACI, total anterior circulation infarct.

### 4.3 Predictors of Mortality Following First‐Ever Acute Ischemic Stroke: Multivariable Model

After adjusting for confounders, the multivariable analysis revealed significant predictors of mortality risks, as shown in Table 3. Factors associated with increased mortality risk include age (adjusted odds ratio [aOR] 1.06 [95% CI 1.03, 1.10; *p *< 0.001]), NIHSS score (aOR 1.08, 95% CI 1.02, 1.13; *p *= 0.004), and diabetes (aOR 2.29, 95% CI 1.20, 4.37; *p *= 0.01). Conversely, factors significantly associated with decreased risk of mortality include antiplatelet therapy within 48 h. (aOR 0.40, 95% CI 0.19, 0.81; *p *= 0.01), dysphagia screening (aOR 0.30, 95% CI 0.15, 0.61; *p *= 0.001), antiplatelet therapy upon discharge (aOR 0.17, 95% CI 0.08, 0.35; *p *< 0.001), lipid‐lowering therapy (aOR 0.37, 95% CI 0.17, 0.82; *p *= 0.01), stroke education (aOR 0.02, 95% CI 0.01, 0.05; *p *< 0.001), and rehabilitation (aOR 0.08, 95% CI 0.04, 0.16; *p *< 0.001).

**Table 3:** Predictors of mortality following first‐ever acute ischemic stroke: multivariable model.

Variables	β (SE)	aOR (95% CI)	*p*‐values
Age	0.06 (0.02)	1.06 (1.03–1.10)	< 0.001
NIHSS score	0.07 (0.03)	1.08 (1.02–1.13)	0.004
Risk factors			
Diabetes	0.83 (0.33)	2.29 (1.20–4.37)	0.01
KPIs adherence			
Antiplatelet in 48 h.	−0.93 (0.37)	0.40 (0.19–0.81)	0.01
Dysphagia screening	−1.19 (0.36)	0.30 (0.15–0.61)	0.001
Antiplatelet upon discharge	−1.76 (0.37)	0.17 (0.08–0.35)	< 0.001
Lipid‐lowering therapy	−0.99 (0.40)	0.37 (0.17–0.82)	0.01
Stroke education	−3.77 (0.39)	0.02 (0.01–0.05)	< 0.001
Rehabilitation	−2.54 (0.36)	0.08 (0.04–0.16)	< 0.001

*Note:* Statistical significance at *p *< 0.05 using multiple logistic regression.

Abbreviations: aOR, adjusted odds ratio; CI, confidence interval; GCS, Glasgow Coma score; IHD, ischemic heart disease; KPIs, key performance indicators; LACI, Lacunar infarct; mRS, modified Rankin scale; NIHSS, National Institute of Health Stroke Scale; OCSP, Oxfordshire community stroke project; PACI, partial anterior circulation infarct; POCI, posterior circulation infarct; RM, Malaysian Ringgit; SD, standard deviation; TACI, total anterior circulation infarct; SE, standard error; β, regression coefficient.

### 4.4 Model Validation for Predicting Mortality After First‐Ever Acute Ischemic Stroke

**Figure 1:**
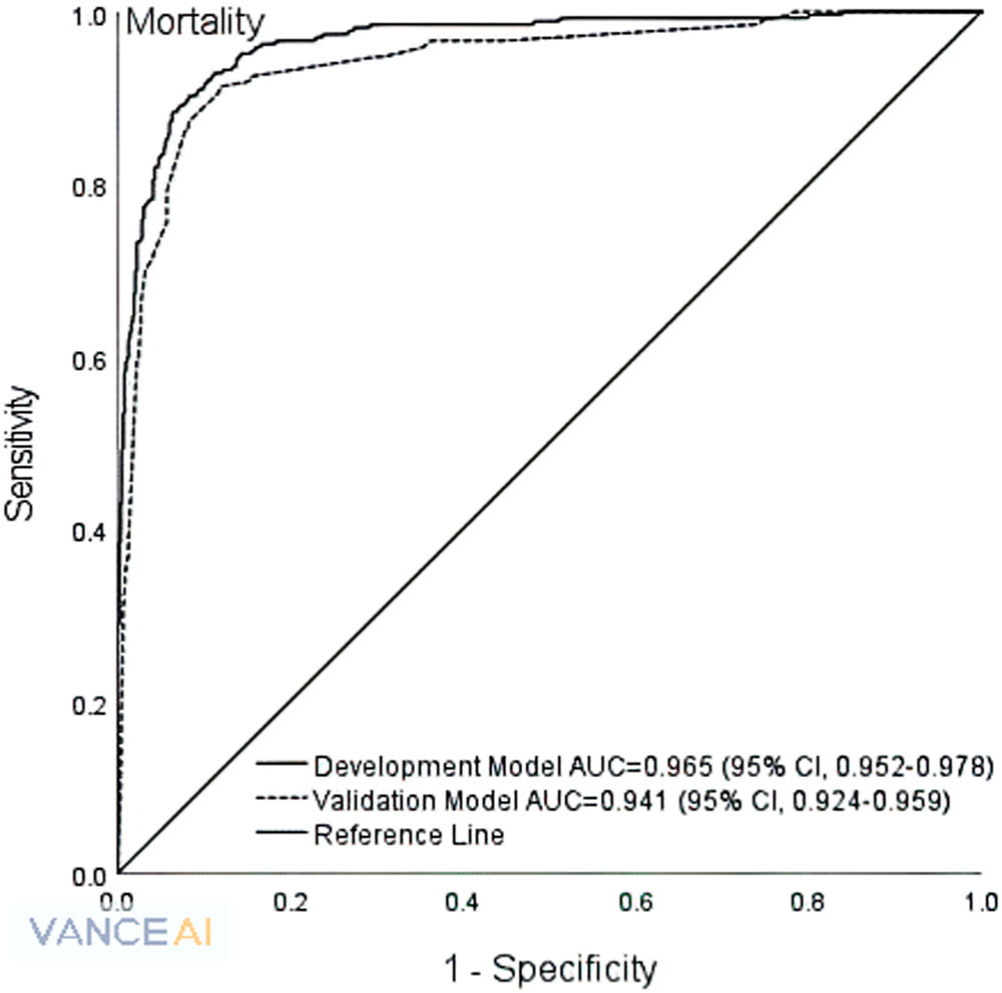
Final model predicting mortality after the first‐ever acute ischemic stroke. AUC, area under the curve; CI, confidence interval.

Both development and validation models demonstrated excellent discrimination (AUC) and calibration (HL *X* ^2^ test), as shown in Table 4 and Figure 1. The development and validation models showed the AUROC was 0.97 (95% CI 0.95, 0.98) versus 0.94 (95% CI 0.92, 0.96), the HL *X* ^2^ was 15.52 (*p *= 0.05) vs 4.35 (*p *= 0.63), the Omnibus *X* ^2^ was 582.49 (*p *< 0.001) vs 142.53 (*p *< 0.001), and *R* ^*2*^ values were 0.80 and 0.74, respectively.

**Table 4:** Model validation for predicting mortality following first‐ever acute ischemic stroke.

Performance measures	Models	
	Development	Validation
Discrimination (AUC)	0.97 (95% CI 0.95–0.98)	0.94 (95% CI 0.92–0.96)
Calibration (H‐L test, *X* ^2^)	15.52 (*p* = 0.05)	4.35 (*p* = 0.63)
Omnibus test (Goodness‐of‐fit, *X* ^2^)	582.49 (*p *< 0.001)	142.53 (*p* < 0.001)
Nagelkerke R‐square (*R* ^*2*^)	0.80	0.74

*Note*: Statistical significance at *p* < 0.05.

Abbreviations: AUC, area under the curve; CI, confidence interval; H‐L, Hosmer‐Lemeshow; X^2^, Chi‐square.

## 5 Discussion

This study developed a validated prognostic model of mortality after a first‐ever acute ischemic stroke using data from the Malaysian National Stroke Registry. The model was excellent in predicting 90‐day mortality post‐stroke with potential clinical application. The overall all‐cause mortality rate was estimated at 29.6% for the study cohort. Significant prognostic indicators of stroke, including mortality, functional disability, complications, or even stroke recurrence, are typically observed within 90 days after stroke [30].

Most of our study population are older male adults. Local studies reported a comparable average age of 60 years in the stroke population [47, 48, 49, 50]. Comparable demographics were reported in Asian studies, including Singapore [51, 52], Thailand [53, 54, 55], Indonesia [56] and slightly higher in China [57, 58, 59] and Taiwan [60, 61]. However, studies from Western countries, particularly the United States, United Kingdom, and Europe, reported an average older age of 70 years [62, 63, 64, 65]. Although stroke can occur at any age, most cases occur among people over the age of 65 [66]. Age is a primary nonmodifiable risk factor for stroke [67]. Older stroke patients face poorer functional recovery, higher mortality, and increased morbidity [67]. The patient's age significantly modifies the role of gender in ischemic stroke [68]. At an early age, the burden of stroke is higher in men with poorer functional outcomes [69]. However, the stroke burden is higher for elderly women, likely due to longer lifespans, hormonal differences, and lifestyle factors [67].

In this study, the distribution of stroke subtypes according to the OCSP classification was majorly LACI. The higher number of patients with LACI and lower with TACI are consistent with similar studies from China [61, 70]. However, it is essential to acknowledge that the distribution of stroke subtypes may vary based on factors such as the study environment and first‐time or recurrent stroke [71]. The GCS score assesses the level of patient consciousness [72]. The present study recorded the average GCS score as mild to moderate. Previous studies on acute ischemic stroke have reported comparable average GCS scores, with more patients presenting with mild scores [47]. Moreover, the higher number of mild GCS scores observed may be attributed to a higher prevalence of the LACI subtype, which typically presents with milder symptoms [71]. Most of the patients in this study had mild‐moderate stroke, assessed using the NIHSS score, a tool that quantifies the severity of stroke symptoms. Similar studies reported comparable NIHSS scores, with most patients presenting with mild‐moderate stroke upon admission [73, 74]. The risk factors identified in this study were majorly hypertension and diabetes, followed by cigarette smoking and hyperlipidemia. Previous studies showed comparable results, with hypertension, diabetes and hyperlipidemia consistently predominant [70, 73, 74]. These risk factors were prevalent in many Asian studies [4, 53, 75]. Specifically, a retrospective study of patients with acute ischemic stroke reported stroke risk factors, predominantly hypertension, followed by diabetes and dyslipidemia in Taiwan [60]. The ability to identify and target at‐risk groups is critical for effective and timely stroke prevention or treatment.

This study developed a validated prognostic model that could reliably predict 90‐day mortality following a first‐ever acute ischemic stroke using the combination of patients’ sociodemographics and clinical and treatment guideline variables. The final prognostic model showed excellent discrimination (AUC, 0.941) and calibration (*X* ^2^, *p *= 0.630). These results suggest that both models had good discriminatory power and were well‐calibrated, suggesting that the model could accurately distinguish between patients with or without the risk of mortality outcome after 90 days post‐stroke. Age, NIHSS score, diabetes, and treatment variables emerged as significant predictors of mortality in the final model. A previous study showed that the 30‐day fatality rate rises from 9% in those aged 65%–74% to 13.1% for those aged 74–84 and reaches 23% for those over 85 years [76]. Additionally, another study reported that 28% of patients over 80 died within 90 days, compared to 13% under 80 age group [77]. Adherence to the stroke treatment guidelines, including antiplatelet therapy within 48 h., dysphagia screening, antiplatelet therapy upon discharge, lipid‐lowering therapy, stroke education, and rehabilitation, were demonstrated to decrease the risk of death. These findings are consistent with previous research highlighting the importance of adherence to evidence‐based guidelines in improving outcomes after stroke [78, 79].

It is worth comparing the results of this study with those of previous studies that have developed and validated prediction models for mortality following stroke. For example, a study by Xian et al. (2016) developed and validated a prediction model for inpatient mortality among acute ischemic stroke patients [80]. The study was from a single center, reported a lower AUC of 0.88 for the validation model predicting only in‐hospital mortality, and included a few variables. However, our study was a robust multicentered study that included several relevant variables, including treatments, to predict 90‐day mortality and had a higher AUC (0.941), suggesting higher, more accuracy and sensitivity. This fact also justifies not aiming at a patient‐based model for predicting the risk of mortality but using variables that are routinely available during acute stroke management and when the first prediction of the fate of the stroke patient is often requested before discharge decisions. Along these lines, the prognostic model could help in identifying patients who need special intervention, including adequate monitoring during the acute phase of ischemic stroke. It could also assist in the decision process of stroke unit triage and inform counseling patients and their relatives about the risk of mortality posthospital discharge.

The study has several strengths and limitations to consider when interpreting the findings. One key strength is using a large, nationally representative sample of stroke patients prospectively collected from multiple stroke centers, which mirrors real‐world clinical practice. This enhances the generalizability of the findings to the broader stroke population and increases the reliability of the results. Another notable strength is developing a valid, guideline‐driven prognostic model, which could aid healthcare providers in identifying high‐risk patients and guiding treatment decisions. Also, another key strength of this study is its focus on patients with first‐ever acute ischemic stroke, which helps to create a more homogenous population with consistent baseline clinical characteristics. This approach minimizes potential confounding factors, allowing for more definitive conclusions specific to first‐ever ischemic stroke patients. Given that ischemic stroke is the most common type of stroke, our findings are highly relevant and have the potential to impact a large portion of the stroke population. Accurate outcome prediction in acute ischemic stroke is critical for enabling clinicians to make timely, evidence‐based decisions. The current study highlights the stroke registry's continued relevance as a national registry supporting active initiatives to enhance evidence‐based stroke care and innovative research.

However, one limitation is the reliance on administrative data from a registry. While this provides a large and representative sample, there may be concerns regarding the completeness of the data. Nevertheless, rigorous quality control measures were implemented to ensure data integrity, including peer evaluation by additional research team members. Despite these efforts, caution is advised when interpreting the findings and applying the models to external populations. Future research would benefit from external validation of the developed models to assess their applicability in different settings. Additionally, the specific causes of death (neurological vs. non‐neurological) were not available in this study or previous registry‐based studies, which would have provided valuable insights. Another consideration is that acute ischemic stroke may sometimes be the presenting manifestation of underlying hematological diseases, such as thrombocythemia, polycythemia vera, thrombotic thrombocytopenic purpura, and acute lymphoblastic leukemia [81]. Therefore, there is a need for further differential diagnoses to rule out hematological diseases for all suspected stroke cases. Lastly, our study had an insignificant number of patients aged 85 and above, probably due to the relatively smaller cohort in the current study. This extreme age group could differ in demographic and clinical characteristics, including the prognosis warranting a subgroup analysis in subsequent studies.

## 6 Conclusion

The study developed a validated prognostic model that predicts mortality after a first‐ever acute ischemic stroke. The model showed excellent validation performance and potential clinical utility. The model could aid in predicting post‐stroke outcomes, offering valuable support for patient management and clinical decision‐making. Moreover, the model may be the foundation for developing user‐friendly web‐based risk calculators, facilitating informed clinical decision‐making by healthcare providers, patients, and other stakeholders. Future research could focus on external validation of the model, including other prognostic factors, and exploring predictive models in different populations.

## Author Contributions

**Mustapha Mohammed:** conceptualization, investigation, funding acquisition, writing – original draft, methodology, validation, writing – review and editing, visualization, software, formal analysis, project administration, data curation, resources. **Hadzliana Zainal:** conceptualization, methodology, validation, funding acquisition, writing – original draft, writing – review and editing, supervision, data curation, investigation, visualization, software, formal analysis, project administration, resources. **Siew Chin Ong:** conceptualization, methodology, validation, writing – original draft writing – review and editing, supervision, data curation, investigation, visualization, funding acquisition, software, formal analysis, project administration, resources. **Balamurugan Tangiisuran:** methodology, writing – original draft, writing – review and editing, formal analysis, data curation, investigation, validation, visualization, software, resources, conceptualization, supervision, funding acquisition. **Fatimatuzzahra Abdul Aziz:** investigation; methodology, writing – review and editing, visualization, software, resources. **Norsima N. Sidek:** methodology, writing – review and editing, visualization, resources, software, investigation. **Abubakar Sha'aban:** investigation, methodology, visualization, writing – review and editing, project administration, software, resources. **Umar Idris Ibrahim:** investigation, writing – review and editing, methodology, visualization, software, project administration, resources. **Surajuddeen Muhammad:** methodology, software, investigation, visualization, resources, project administration, writing – review and editing. **Irene Looi:** writing – review and editing, validation, formal analysis, supervision, data curation, visualization, conceptualization, funding acquisition. **Zariah A. Aziz:** writing – review and editing, visualization, validation, data curation, supervision, formal analysis, conceptualization, funding acquisition.

## Conflicts of Interest

The authors declare no conflicts of interest.

## Transparency Statement

The lead author Mustapha Mohammed, Hadzliana Zainal affirms that this manuscript is an honest, accurate, and transparent account of the study being reported; that no important aspects of the study have been omitted; and that any discrepancies from the study as planned (and, if relevant, registered) have been explained.

## Data Availability

The data that support the findings of this study are available on request from the corresponding author. The data are not publicly available due to privacy or ethical restrictions. The data supporting the findings of this study are available from the corresponding author(s) upon reasonable request. All authors have read and approved the final version of the manuscript. MM and HZ had full access to all of the data in this study and take complete responsibility for the integrity of the data and the accuracy of the data analysis.

## References

[1] V. L. Feigin , B. A. Stark , C. O. Johnson , et al., “Global, Regional, and National Burden of Stroke and its Risk Factors, 1990–2019: A Systematic Analysis for the Global Burden of Disease Study 2019,” Lancet Neurology 20, no. 10 (2021): 795–820. 34487721 10.1016/S1474-4422(21)00252-0 PMC8443449

[2] S. Rajsic , H. Gothe , H. H. Borba , et al., “Economic Burden of Stroke: A Systematic Review on Post‐Stroke Care,” European Journal of Health Economics 20, no. 1 (2019): 107–134. 10.1007/s10198-018-0984-0 29909569

[3] E. J. Benjamin , S. S. Virani , C. W. Callaway , et al., “Heart Disease and Stroke Statistics‐2018 Update: A Report From the American Heart Association,” Circulation 137, no. 12 (2018): e67–e492. 29386200 10.1161/CIR.0000000000000558

[4] N. Venketasubramanian , B. W. Yoon , J. Pandian , and J. C. Navarro , “Stroke Epidemiology in South, East, and South‐East Asia: A Review,” Journal of Stroke 19, no. 3 (2017): 286–294. 29037005 10.5853/jos.2017.00234 PMC5647629

[5] P. K. Chia , N. A. Mohamad , and L. N. I. Mat , et al., “Regional Emergency Stroke Quick‐Response (RESQ) Network: A Proposed Paradigm of Malaysia Stroke Care Services,” Malaysian Journal of Medicine and Health Sciences 16, no. 4 (2020): 353–361.

[6] K. S. Tan and N. Venketasubramanian , “Stroke Burden in Malaysia,” Cerebrovascular Diseases Extra 12, no. 2 (2022): 58–62. 35325896 10.1159/000524271 PMC9149343

[7] R.‐J. Singh , S. Chen , A. Ganesh , and M. D. Hill , “Long‐Term Neurological, Vascular, and Mortality Outcomes After Stroke,” International Journal of Stroke 13, no. 8 (2018): 787–796. 30160619 10.1177/1747493018798526

[8] S. Koton , A. L. C. Schneider , W. D. Rosamond , et al., “Stroke Incidence and Mortality Trends in US Communities, 1987 to 2011,” Journal of the American Medical Association 312, no. 3 (2014): 259–268. 25027141 10.1001/jama.2014.7692

[9] F. Jaya , M. N. Win , M. R. Abdullah , M. R. Abdullah , and J. M. Abdullah , “Stroke Patterns in Northeast Malaysia: A Hospital‐Based Prospective Study,” Neuroepidemiology 21, no. 1 (2001): 28–35. 10.1159/000048611 11744823

[10] W. Y. Hwong , S. H. Ang , M. L. Bots , et al., “Trends of Stroke Incidence and 28‐Day All‐Cause Mortality After a Stroke in Malaysia: A Linkage of National Data Sources,” Global Heart 16, no. 1 (2021): 39. 34211825 10.5334/gh.791 PMC8162294

[11] M. El Hajj , R. Abdo , S. Assaf , and N. Lahoud , “Stroke Management in Developing Countries,” in Handbook of Medical and Health Sciences in Developing Countries: Education, Practice, and Research (Springer, 2023), 1–31.

[12] M. H. Murad , Clinical Practice Guidelines: A primer on Development and Dissemination. Mayo Clinic Proceedings (Elsevier, 2017), 423–433. 10.1016/j.mayocp.2017.01.001 28259229

[13] Z. A. Aziz , Y. Y. L. Lee , B. A. Ngah , et al., “Acute Stroke Registry Malaysia, 2010‐2014: Results From The National Neurology Registry,” Journal of Stroke and Cerebrovascular Diseases 24, no. 12 (2015): 2701–2709. 26338106 10.1016/j.jstrokecerebrovasdis.2015.07.025

[14] A. A. Al‐Temimi , S. H. Gan , and C. S. Selvaraj , “Effective Application of Pharmacotherapy Strategy for Hospitalized Ischemic Stroke Patients in Malaysia,” Journal of Medical Care Research and Review 3, no. 8 (2020): 404–412.

[15] N. E. McMahon , M. Bangee , V. Benedetto , et al., “Etiologic Workup in Cases of Cryptogenic Stroke: A Systematic Review of International Clinical Practice Guidelines,” Stroke 51, no. 5 (2020): 1419–1427. 32279620 10.1161/STROKEAHA.119.027123 PMC7185056

[16] L. Chen , “Overview of Clinical Prediction Models,” Annals of Translational Medicine 8, no. 4 (2020): 71. 32175364 10.21037/atm.2019.11.121 PMC7049012

[17] M. Mohammed , H. Zainal , and S. C. Ong , “Trends in Clinical Prediction Models of Stroke Outcomes Research: A Bibliometric Analysis,” ASM Science Journal 16, no. 2021 (2022): 1–10.

[18] M. Fahey , E. Crayton , C. Wolfe , and A. Douiri , “Clinical Prediction Models for Mortality and Functional Outcome Following Ischemic Stroke: A Systematic Review and Meta‐Analysis,” PLoS One 13, no. 1 (2018): e0185402. 29377923 10.1371/journal.pone.0185402 PMC5788336

[19] R. B. D'Agostino , R. S. Vasan , M. J. Pencina , et al., “General Cardiovascular Risk Profile for Use in Primary Care,” Circulation 117, no. 6 (2008): 743–753. 18212285 10.1161/CIRCULATIONAHA.107.699579

[20] J. Hippisley‐Cox , C. Coupland , Y. Vinogradova , J. Robson , M. May , and P. Brindle , “Derivation and Validation of QRISK, a New Cardiovascular Disease Risk Score for the United Kingdom: Prospective Open Cohort Study,” BMJ 335, no. 7611 (2007): 136. 17615182 10.1136/bmj.39261.471806.55 PMC1925200

[21] F. Roques , P. Michel , and A. R. Goldstone , “The Logistic Euroscore,” European Heart Journal 24, no. 9 (2003): 882–883. 10.1016/s0195-668x(02)00799-6 12727160

[22] M. Fahey , A. Rudd , Y. Béjot , C. Wolfe , and A. Douiri , “Development and Validation of Clinical Prediction Models for Mortality, Functional Outcome and Cognitive Impairment After Stroke: A Study Protocol,” BMJ Open 7, no. 8 (2017): e014607. 10.1136/bmjopen-2016-014607 PMC5724146 28821511

[23] M. E. Shipe , S. A. Deppen , F. Farjah , and E. L. Grogan , “Developing Prediction Models for Clinical Use Using Logistic Regression: An Overview,” Journal of Thoracic Disease 11, no. Suppl 4 (2019): S574–S584. 31032076 10.21037/jtd.2019.01.25 PMC6465431

[24] G. S. Collins , J. B. Reitsma , D. G. Altman , and K. G. M. Moons , “Transparent Reporting of a Multivariable Prediction Model for Individual Prognosis or Diagnosis (Tripod) the Tripod Statement,” Circulation 131, no. 2 (2015): 211–219. 25561516 10.1161/CIRCULATIONAHA.114.014508 PMC4297220

[25] J. P. Vandenbroucke , E. von Elm , D. G. Altman , et al., “Strengthening the Reporting of Observational Studies in,” International Journal of Surgery (London, England) 12, no. 12 (2014): 1500–1524. 25046751 10.1016/j.ijsu.2014.07.014

[26] M. Assel , D. Sjoberg , A. Elders , et al., “Guidelines for Reporting of Statistics for Clinical Research in Urology,” Journal of Urology 201, no. 3 (2019): 595–604. 30633111 10.1097/JU.0000000000000001 PMC6600813

[27] M. Mohammed , H. Zainal , and B. T. Angiisuran , et al., “Impact of Adherence to Key Performance Indicators on Mortality Among Patients Managed for Ischemic Stroke,” Pharmacy Practice (Granada) 18, no. 1 (2020): 1760. 10.18549/PharmPract.2020.1.1760 PMC7092711 32256900

[28] M. Lee , Y.‐L. Wu , and B. Ovbiagele , “Trends in Incident and Recurrent Rates of First‐Ever Ischemic Stroke in Taiwan Between 2000 and 2011,” Journal of Stroke 18, no. 1 (2016): 60–65. 26687123 10.5853/jos.2015.01326 PMC4747065

[29] J. O. Cerasuolo , J. Mandzia , L. E. Cipriano , et al., “Intravenous Thrombolysis After First‐Ever Ischemic Stroke and Reduced Incident Dementia Rate,” Stroke 53, no. 4 (2022): 1170–1177. 34965738 10.1161/STROKEAHA.121.034969

[30] N. Jampathong , M. Laopaiboon , S. Rattanakanokchai , and P. Pattanittum , “Prognostic Models for Complete Recovery in Ischemic Stroke: A Systematic Review and Meta‐Analysis,” BMC Neurology 18, no. 1 (2018): 26. 29523104 10.1186/s12883-018-1032-5 PMC5845155

[31] R. Pujadas Capmany , A. Arboix , R. Casañas‐Muñoz , and N. Anguera‐Ferrando , “Specific Cardiac Disorders in 402 Consecutive Patients With Ischaemic Cardioembolic Stroke,” International Journal of Cardiology 95, no. 2–3 (2004): 129–134. 15193810 10.1016/j.ijcard.2003.02.007

[32] B. A. Mateen , M. Horton , and E. D. Playford , “Psychometric Analysis of the Glasgow Coma Scale and its Sub‐Scale Scores in a National Retrospective Cohort of Patients With Traumatic Injuries,” PLoS One 17, no. 6 (2022): e0268527. 35675316 10.1371/journal.pone.0268527 PMC9176762

[33] J. de Andrade , J. P. Mohr , F. B. Timbó , et al., “Oxfordshire Community Stroke Project Classification: A Proposed Automated Algorithm,” European Stroke Journal 6, no. 2 (2021): 160–167. 34414291 10.1177/23969873211012136 PMC8370065

[34] P. Lyden , “Using The National Institutes of Health Stroke Scale: A Cautionary Tale,” Stroke 48, no. 2 (2017): 513–519. 28077454 10.1161/STROKEAHA.116.015434

[35] A. N. Richter and T. M. Khoshgoftaar , “A Review of Statistical and Machine Learning Methods for Modeling Cancer Risk Using Structured Clinical Data,” Artificial Intelligence in Medicine 90 (2018): 1–14. 30017512 10.1016/j.artmed.2018.06.002

[36] G.‐W. Sun , T. L. Shook , and G. L. Kay , “Inappropriate Use of Bivariable Analysis to Screen Risk Factors for Use in Multivariable Analysis,” Journal of Clinical Epidemiology 49, no. 8 (1996): 907–916. 8699212 10.1016/0895-4356(96)00025-x

[37] M. Mustapha , B. K. Lawal , A. Sha'aban , et al., “Factors Associated With Acceptance of COVID‐19 Vaccine Among University Health Sciences Students in Northwest Nigeria,” PLoS One 16, no. 11 (2021): e0260672. 34843594 10.1371/journal.pone.0260672 PMC8629299

[38] M. Mohammed , S. Muhammad , F. Z. Mohammed , et al., “Risk Factors Associated With Mortality Among Patients With Novel Coronavirus Disease (COVID‐19) in Africa,” Journal of Racial and Ethnic Health Disparities 8 (2021): 1267–1272. 33051749 10.1007/s40615-020-00888-3 PMC7553376

[39] D. A. Walker and T. J. Smith , “JMASM36: Nine Pseudo R^2 Indices for Binary Logistic Regression Models (SPSS),” Journal of Modern Applied Statistical Methods 15, no. 1 (2016): 848–854.

[40] A. E. Ivanescu , P. Li , B. George , et al., “The Importance of Prediction Model Validation and Assessment in Obesity and Nutrition Research,” International Journal of Obesity 40, no. 6 (2016): 887–894. 26449421 10.1038/ijo.2015.214 PMC4826636

[41] E. W. Steyerberg , A. J. Vickers , N. R. Cook , et al., “Assessing the Performance of Prediction Models: A Framework for Traditional and Novel Measures,” Epidemiology 21, no. 1 (2010): 128–138. 20010215 10.1097/EDE.0b013e3181c30fb2 PMC3575184

[42] K. G. M. Moons , A. P. Kengne , D. E. Grobbee , et al., “Risk Prediction Models: Ii. External Validation, Model Updating, and Impact Assessment,” Heart 98, no. 9 (2012): 691–698. 22397946 10.1136/heartjnl-2011-301247

[43] K. Han , K. Song , and B. W. Choi , “How to Develop, Validate, and Compare Clinical Prediction Models Involving Radiological Parameters: Study Design and Statistical Methods,” Korean Journal of Radiology 17, no. 3 (2016): 339–350. 27134523 10.3348/kjr.2016.17.3.339 PMC4842854

[44] R. D. Riley , J. Ensor , and K. I. E. Snell , “Calculating the Sample Size Required for Developing a Clinical Prediction Model,” BMJ 2020, no. 368 (2020): m441. 10.1136/bmj.m441 32188600

[45] T. A. Lang and D. G. Altman , “Statistical Analyses and Methods in the Published Literature: The SAMPL Guidelines,” Guidelines for Reporting Health Research: A User's Manual 25 (2014): 264–274.

[46] R. Pp , “Human Experimentation. Code of Ethics of the World Medical Association,” Declaration of Helsinki. British Medical Journal 2, no. 5402 (1964): 177. 14150898 10.1136/bmj.2.5402.177 PMC1816102

[47] N. Wan‐Arfah , H. M. Hafiz , N. N. Naing , M. Muzaimi , and H. G. M. Shetty , “Short‐Term and Long‐Term Survival Probabilities Among First‐Ever Ischaemic and Haemorrhagic Stroke Patients at a Hospital in the Suburban East Coast of Peninsular Malaysia,” Health Science Reports 1, no. 2 (2018): e27. 30623059 10.1002/hsr2.27 PMC6266517

[48] H. C. Loh , et al., “Socio‐Demographics and Clinical Characteristics Affecting Pre‐Hospital Delays in Acute Stroke Patients: A 6‐year Registry Study From a Malaysian Stroke Hospital. Neurology,” Asia 25 (2020): 3.

[49] X. W. Chen , M. N. Shafei , Z. A. Aziz , N. N. Sidek , and K. I. Musa , “Trends in Stroke Outcomes at Hospital Discharge in First‐Ever Stroke Patients: Observations From the Malaysia National Stroke Registry (2009–2017),” Journal of the Neurological Sciences 401 (2019): 130–135. 31000206 10.1016/j.jns.2019.04.015

[50] H. C. Loh , K. Ganasegeran , Y. F. Lim , and I. Looi , “Comparison Among Demographics, Risk Factors, Clinical Manifestations, and Outcomes of Stroke Subtypes: Findings From a Malaysian Stroke‐Ready Hospital,” Neurology Asia 27, no. 1 (2022): 25–34.

[51] S.‐H. Yeo , M. P. H. Toh , S. H. Lee , R. C. Seet , L. Y. Wong , and W. P. Yau , “Temporal Trends and Patient Characteristics Associated With Drug Utilisation After First‐Ever Stroke: Insights From Chronic Disease Registry Data in Singapore,” Annals of the Academy of Medicine, Singapore 49, no. 3 (2020): 137–154. 32301477

[52] H. C. Loh , J. N. Kwan , and S. N. L. Ramlee , “The Impact of Sociodemographic Variables on Risk Factors Associated With Ischaemic Stroke Patients by Age Group,” Journal Of Cardiovascular, Neurovascular & Stroke 4, no. 1 (2022): 8–21.

[53] C. Samuthpongtorn , T. Jereerat , and N. C. Suwanwela , “Stroke Risk Factors, Subtypes and Outcome in Elderly Thai Patients,” BMC Neurology 21, no. 1 (2021): 322. 34416866 10.1186/s12883-021-02353-y PMC8377861

[54] N. Kasemsap , N. Vorasoot , K. Kongbunkiat , U. Peansukwech , S. Tiamkao , and K. Sawanyawisuth , “Impact of Intravenous Thrombolysis on Length of Hospital Stay in Cases of Acute Ischemic Stroke,” Neuropsychiatric Disease and Treatment 14 (2018): 259–264. 29386899 10.2147/NDT.S151836 PMC5767097

[55] W. Chantkran , J. Chaisakul , and R. Rangsin , “Prevalence of and Factors Associated With Stroke in Hypertensive Patients in Thailand From 2014 to 2018: A Nationwide Cross‐Sectional Study,” Scientific Reports 11, no. 1 (2021): 17614. 34475463 10.1038/s41598-021-96878-4 PMC8413271

[56] R. T. Pinzon and F. Buwana , “The Comparison of Risk Factor Between the Young Adult and Elderly Onset of Ischemic Stroke,” Journal of Clinical Medicine of Kazakhstan 2, no. 48 (2018): 28–32.

[57] M. Liu , M. Yan , Y. Guo , et al., “Acute Ischemic Stroke at High Altitudes in China: Early Onset and Severe Manifestations,” Cells 10, no. 4 (2021): 809. 33916503 10.3390/cells10040809 PMC8067425

[58] Y. Wang , X. Liao , C. Wang , et al., “Impairment of Cognition and Sleep After Acute Ischaemic Stroke or Transient Ischaemic Attack in Chinese Patients: Design, Rationale and Baseline Patient Characteristics of a Nationwide Multicentre Prospective Registry,” Stroke and Vascular Neurology 6, no. 1 (2021): 139–144. 32665365 10.1136/svn-2020-000359 PMC8005906

[59] D. Kong , X. Liu , H. Lian , et al., “Analysis of Hospital Charges of Inpatients With Acute Ischemic Stroke in Beijing, China, 2012–2015,” Neuroepidemiology 50, no. 1–2 (2018): 63–73. 29421788 10.1159/000484212

[60] M.‐T. Hsieh , C. Y. Hsieh , T. T. Tsai , and S. F. Sung , “Validation of Stroke Risk Factors in Patients With Acute Ischemic Stroke, Transient Ischemic Attack, or Intracerebral Hemorrhage on Taiwan's National Health Insurance Claims Data,” Clinical Epidemiology 14 (2022): 327–335. 35330593 10.2147/CLEP.S353435 PMC8938165

[61] C.‐F. Tsai , C. L. M. Sudlow , N. Anderson , and J. S. Jeng , “Variations of Risk Factors for Ischemic Stroke and its Subtypes in Chinese Patients in Taiwan,” Scientific Reports 11, no. 1 (2021): 9700. 33958684 10.1038/s41598-021-89228-x PMC8102638

[62] O. O. Seminog , P. Scarborough , F. L. Wright , M. Rayner , and M. J. Goldacre , “Determinants of the Decline in Mortality From Acute Stroke in England: Linked National Database Study of 795869 Adults,” BMJ 365 (2019): l1778. 31122927 10.1136/bmj.l1778 PMC6529851

[63] K. Kotfis , M. Bott‐Olejnik , A. Szylińska , M. Listewnik , and I. Rotter , “Characteristics, Risk Factors and Outcome of Early‐Onset Delirium in Elderly Patients With First Ever Acute Ischemic,” Clinical Interventions in Aging 14 (2019): 1771–1782. 31695347 10.2147/CIA.S227755 PMC6814355

[64] L. A. Sposato , M. Lam , B. Allen , S. Z. Shariff , and G. Saposnik , “First‐Ever Ischemic Stroke and Incident Major Adverse Cardiovascular Events in 93627 Older Women and Men,” Stroke 51, no. 2 (2020): 387–394. 31914883 10.1161/STROKEAHA.119.028066

[65] S. K. Anand , W. J. Benjamin , A. R. Adapa , et al., “Trends in Acute Ischemic Stroke Treatments and Mortality in the United States From 2012 to 2018,” Neurosurgical Focus 51, no. 1 (2021): E2. 10.3171/2021.4.FOCUS21117 34198248

[66] E. J. Benjamin , M. J. Blaha , S. E. Chiuve , et al., “Heart Disease and Stroke Statistics—2017 Update: A Report From the American Heart Association,” Circulation 135, no. 10 (2017): e146–e603. 28122885 10.1161/CIR.0000000000000485 PMC5408160

[67] M. Roy‐O'Reilly and L. D. McCullough , “Age and Sex Are Critical Factors in Ischemic Stroke Pathology,” Endocrinology 159, no. 8 (2018): 3120–3131. 30010821 10.1210/en.2018-00465 PMC6963709

[68] R. Weber , C. Krogias , J. Eyding , et al., “Age and Sex Differences in Ischemic Stroke Treatment in a Nationwide Analysis of 1.11 Million Hospitalized Cases,” Stroke 50, no. 12 (2019): 3494–3502. 31623547 10.1161/STROKEAHA.119.026723

[69] S. H. Bots , S. A. Peters , and M. Woodward , “Sex Differences in Coronary Heart Disease and Stroke Mortality: A Global Assessment of the Effect of Ageing Between 1980 and 2010,” BMJ Global Health 2, no. 2 (2017): e000298. 10.1136/bmjgh-2017-000298 PMC5435266 28589033

[70] Y. Lu , C. Zhuoga , H. Jin , et al., “Characteristics of Acute Ischemic Stroke in Hospitalized Patients in Tibet: A Retrospective Comparative Study,” BMC Neurology 20, no. 1 (2020): 380. 33087079 10.1186/s12883-020-01957-0 PMC7576739

[71] C. Juli , H. Heryaman , Arnengsih , et al., “The Number of Risk Factors Increases the Recurrence Events in Ischemic Stroke,” European Journal of Medical Research 27, no. 1 (2022): 138. 35918760 10.1186/s40001-022-00768-y PMC9344667

[72] M. Khoshfetrat , M. A. Yaghoubi , B. M. K. Hosseini , and R. Farahmandrad , “The Ability of GCS, FOUR, and APACHE II in Predicting the Outcome of Patients With Traumatic Brain Injury: A Comparative Study,” Biomedical Research and Therapy 7, no. 2 (2020): 3614–3621.

[73] M. M. Wirtz , P. Hendrix , O. Goren , et al., “Predictor of 90‐day Functional Outcome After Mechanical Thrombectomy for Large Vessel Occlusion Stroke: NIHSS Score of 10 or Less at 24 Hours,” Journal of Neurosurgery 134, no. 1 (2021): 115–121. 31860816 10.3171/2019.10.JNS191991

[74] M. Freitas‐Silva , R. Medeiros , and J. P. L. Nunes , “Risk Factors Among Stroke Subtypes and its Impact on the Clinical Outcome of Patients of Northern Portugal Under Previous Aspirin Therapy,” Clinical Neurology and Neurosurgery 203 (2021): 106564. 33714797 10.1016/j.clineuro.2021.106564

[75] X. Xia , W. Yue , B. Chao , et al., “Prevalence and Risk Factors of Stroke in the Elderly in Northern China: Data From The National Stroke Screening Survey,” Journal of Neurology 266, no. 6 (2019): 1449–1458. 30989368 10.1007/s00415-019-09281-5 PMC6517347

[76] A. S. Go , D. Mozaffarian , V. L. Roger , et al., “Heart Disease and Stroke statistics—2014 Update: A Report From the American Heart Association,” Circulation 129, no. 3 (2014): e28–e292. 24352519 10.1161/01.cir.0000441139.02102.80 PMC5408159

[77] L. Bentsen , L. Christensen , A. Christensen , and H. Christensen , “Outcome and Risk Factors Presented in Old Patients Above 80 Years of Age Versus Younger Patients After Ischemic Stroke,” Journal of Stroke and Cerebrovascular Diseases 23, no. 7 (2014): 1944–1948. 24794945 10.1016/j.jstrokecerebrovasdis.2014.02.002

[78] J. H. Lichtman , S. B. Jones , Y. Wang , E. Watanabe , E. Leifheit‐Limson , and L. B. Goldstein , “Outcomes After Ischemic Stroke for Hospitals With and Without Joint Commission–Certified Primary Stroke Centers,” Neurology 76, no. 23 (2011): 1976–1982. 21543736 10.1212/WNL.0b013e31821e54f3 PMC3109877

[79] G. C. Fonarow , M. J. Reeves , E. E. Smith , et al., “Characteristics, Performance Measures, and In‐Hospital Outcomes of the First One Million Stroke and Transient Ischemic Attack Admissions in Get With the Guidelines‐Stroke,” Circulation. Cardiovascular Quality and Outcomes 3, no. 3 (2010): 291–302. 20177051 10.1161/CIRCOUTCOMES.109.921858

[80] Y. Xian , J. J. Federspiel , M. Grau‐Sepulveda , et al., “Risks and Benefits Associated With Prestroke Antiplatelet Therapy Among Patients With Acute Ischemic Stroke Treated With Intravenous Tissue Plasminogen Activator,” JAMA Neurology 73, no. 1 (2016): 50–59. 26551916 10.1001/jamaneurol.2015.3106

[81] A. Arboix and C. Besses , “Cerebrovascular Disease as the Initial Clinical Presentation of Haematological Disorders,” European Neurology 37, no. 4 (1997): 207–211. 9208259 10.1159/000117444

